# Celtic Invasive Plants database

**DOI:** 10.1038/s41597-026-07636-y

**Published:** 2026-06-24

**Authors:** Claudia González-Toral, Luz Madrazo-Frías, Aránzazu Estrada Fernández, Ricardo López-Alonso, Mauro Sanna, Candela Cuesta, Eduardo Cires, Juan Viruel

**Affiliations:** 1 Royal Botanic Gardens, Kew, Richmond, TW9 3AE UK; 2 Institute of Natural Resources and Territorial Planning (INDUROT), Campus de Mieres, Spain; 3 Department of Organisms and Systems Biology, University of Oviedo, C/. Catedrático Rodrigo Uría s/n, 33071 Oviedo, Spain; 4 Escuela Politécnica Superior de Huesca, Universidad de Zaragoza, Crta. Cuarte sn, 22071 Huesca, Spain; 5 Institute for Biocomputation and Physics of Complex Systems (BIFI) of Universidad de Zaragoza, Zaragoza, Spain

## Abstract

Alien Invasive Species (AIS) are among the principal threats to humanity due to their substantial ecological, social, and economic impacts. International efforts to harmonize national AIS checklists and databases is often hindered by fragmented data across multiple platforms. The Celtic Fringe, a biogeographically coherent unit within the European Atlantic Floristic Region, includes part of the Iberian Peninsula, France and the British Isles, and is notable for its extensively documented flora. Here, we present the first unified AIS checklist and georeferenced occurrence database for the entire Celtic Fringe, with occurrences mapped to a 10 × 10 km UTM grid resolution. Occurrence data were aggregated from public datasets, while the harmonized AIS checklist of 271 taxa was developed through the integration of national and international sources. The resulting database comprises 164,974 occurrences, each enriched with taxonomic, floristic, and administrative metadata to facilitate use across multiple geographic and governance levels. This harmonized and standardised resource is designed to support AIS management at local, national, and transnational scales, while facilitating conservation planning and research on invasion dynamics.

## Background & Summary

Alien Invasive Species (AIS) are among the most urgent global threats to biodiversity, human well-being, and socio-economic stability due to their extensive ecological, health-related, and economic impacts. The accelerating globalisation of trade, transport, and tourism has facilitated both the unintentional and deliberate introduction of non-native species across ecosystems worldwide. Such introductions can trigger biological invasions, often resulting in irreversible consequences^1–3^. AIS occur across all major taxonomic groups, with terrestrial invertebrates and vascular plants representing the most abundant and impactful invaders^4–6^. Their spread and impacts are further amplified by anthropogenic disturbances and climate change, which together intensify their threat at a global scale^7^. Moreover, global AIS richness continues to rise with no signs of saturation. Notably, over one-third of all first records have been reported since the 1970s^8^. Recent estimates suggest that even a relatively small subset of AIS imposes a global economic burden of 2,215 billion USD, with some species also causing cascading social consequences and profound environmental alterations^1,2,9,10^.

The European Atlantic Floristic region, characterized by high rainfall, an oceanic climate, and distinct floristic affinities, includes several subprovinces along western Europe. These extend across parts of the Iberian Peninsula, France, and the British Isles^11–17^. Within this region, the Celtic Fringe has long been recognized as the climatic and floristic core^12,15,18–21^. The Celtic Fringe comprises territories from six countries (Portugal, Spain, France, United Kingdom, the Republic of Ireland and Denmark (i.e., Faroe Islands)), and is home to some of the most extensively documented floras in the world^13,19,22,23^. However, because national checklists often cover multiple floristic regions (e.g., Mediterranean and continental), species recognized as invasive at the national scale may not necessarily be invasive within the Celtic Fringe. This highlights the need for regional harmonization.

In this study, we present the first integrated AIS checklist and spatial database for the Celtic Fringe. We compiled data from national and international checklists and occurrence records, which were standardized and mapped onto a unified 10 × 10 km UTM grid. Each record includes taxonomic, floristic, and administrative metadata, enabling flexible use across geographical and policy-relevant scales. This resource offers a crucial tool for local, national, and transnational management of biological invasions, supports conservation prioritization, and advances our understanding of species distribution patterns under ongoing ecological change. Furthermore, by aligning with frameworks such as the Union List of Invasive Alien Species of Union Concern^24–26^ and the Environmental Impact Classification for Alien Taxa (EICAT)^27^, this work contributes to regulatory decision-making at broad scales.

## Methods

### Area of study: the Celtic Fringe of the European Atlantic Floristic Region

The study focuses on the Celtic Fringe within the European Atlantic Floristic Region, an area characterised by high precipitation and mild temperatures influenced by the Atlantic Ocean^11,12,15,18^. This region is marked by low seasonality and limited temperature variation, conditions that have fostered distinctive floristic assemblages along the western coast of Europe^12–16,28^.

Although the number and delimitation of the subprovinces comprising this region remain debated, four subprovinces (Cantabrian Atlantic, Orocantabrian, French Atlantic, and Britannic) are widely accepted as forming the core of the European Atlantic Floristic Region^12,14–16,19^. The Cantabrian Atlantic subprovince encompasses areas of northern Spain and Portugal as well as Gascogne, Aquitaine, and des Landes in France. The Orocantabrian subprovince includes most of the Cantabrian Range and surrounding areas in northern Spain. The French Atlantic subprovince covers much of northwestern France, including Normandy, Brittany, and the Paris Basin. The Britannic subprovince comprises the British Isles and the Faroe Islands^12,14–16,28^.

**Fig. 1:**
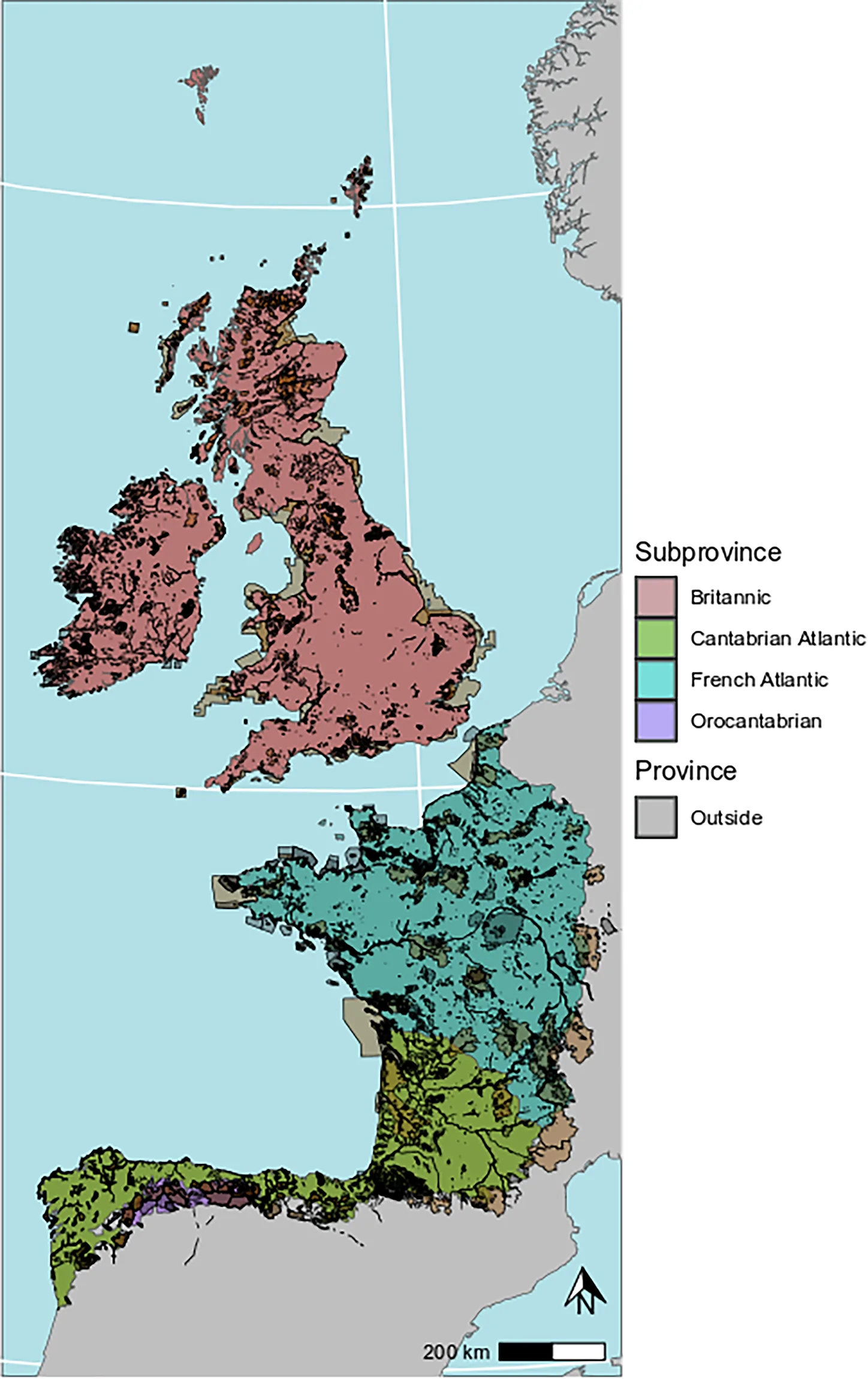
Graphical representation of the different UTM 10 × 10 Km grids conforming the area of interest of this study: the Celtic Fringe of the European Atlantic Floristic Region. The outer boundaries followed European Environment Agency (EEA)^13^, Rivas-Martínez, Penas & Díaz^14^, and Fernández Prieto *et al*.^16^. The boundaries of the four different subprovinces of the the Celtic Fringe– the Cantabrian Atlantic, the Orocantabrian, the French Atlantic and the Britannic — are in accordance to those proposed by Rivas-Martínez, Penas & Díaz^14^, Fernández Prieto *et al*.^16^ and Instituto Geográfico Nacional^30^. These subprovinces have been represented using different colours. Polygons correspond to the National Protected Areas (light brown) and Natura 2000 Protected Areas (light grey).

For this study, the outer delimitation of the Celtic Fringe was established by the boundaries of European Environment Agency (EEA)^13^, Rivas-Martínez, Penas & Díaz^14^, and Fernández Prieto *et al*.^16^. These boundaries encompass territories in Portugal, Spain, France, the United Kingdom, the Republic of Ireland, and Denmark (specifically the Faroe Islands). They were chosen to include transitional zones influenced by Mediterranean, Central European, and Alpino-Caucasian elements^16,20,28^, thereby capturing the complex bioclimatic gradients of the western Atlantic region. Importantly, the chosen delimitation includes both insular and continental areas, allowing the database to reflect the full spectrum of biogeographic and administrative diversity present in this core Atlantic floristic unit^12,14–16,28^ (see Fig. 1).

Nevertheless, the boundaries of this subregion remain contentious, particularly regarding southwestern and southeastern transitional areas where mixed climatic conditions (e.g., Temperate and Mediterranean) and shared floristic elements coexist^15,16,18,21,29^. In this context, we adopted the outer delimitations proposed by European Environment Agency (EEA)^13^, Rivas-Martínez, Penas & Díaz^14^ and Fernández Prieto *et al*.^16^. By including both French and Iberian boundaries, we account for areas in contact with the Western Mediterranean bioclime and Alpino-Caucasian and Central European Floras, while also integrating insular and mainland continental territories^14,16,18,20^. This sensu lato approach to the outer boundaries includes transitional high-mountain zones with diverse climatic features^28^. Overall, this area encompasses territories belonging to six countries (Portugal, Spain, France, the United Kingdom, Ireland and Denmark (i.e. the Faroe Islands)), five of which are currently members of the European Union (EU).

The inner boundaries of the Celtic Fringe delimiting its subprovinces were establishing following Rivas-Martínez, Penas & Díaz^14^ and the more detailed Cantabrian Atlantic-Orocantabrian boundary of Instituto Geográfico Nacional^30^.

### AIS Checklists sources and preliminary AIS checklist standardization

To generate a unified invasive species checklist for the Celtic Fringe of the European Atlantic Floristic Region, we compiled official national and international checklists from the six constituent countries: Portugal, Spain, France, the United Kingdom, Ireland, and Denmark (i.e., Faroe Islands). Only vascular plant species (Tracheophyta) explicitly labelled as “invasive” were considered. Species considered invasive in other regions, such as Mediterranean or oceanic islands, but not within the Celtic Fringe were excluded.

The official Portuguese invasive species checklist was obtained from the Ministry of Environment and Energetic Transition of the Portuguese Government^31^. The Spanish checklist was compiled from several publications of the Ministry for the Ecological Transition^32–35^. For France, the sources included the “Espèces Introduites Envahissantes” of the “Référentiel taxonomique version 18” (Tax Ref v18) from the Inventaire National du Patrimoine Naturel (INPN)^36^, which compiles the AIS checklists from the different Conservatoire Botanique National as well as updated ministerial orders (arrêtés) from the French Ministry of Ecological Transition (Minitère de la Transition Écologique)^37–39^. We only considered those Tax Ref v18 species officially listed in “France métropolitaine” (i. e. continental France). Data from the United Kingdom were obtained from the Natural England report^40^, the Great Britain Non-Native Species Secretariat (NNSS) Species of Special Concern^41^ and the comprehensive compilation of all the UK checklists provided by the Royal Horticultural Society (RHS)^42,43^. For Ireland, the checklist was retrieved from the National Biodiversity Data Centre of Ireland and included the updates from the Government^44–47^. In the case of the Faroe Islands (Denmark), the two legally binding checklists (the Union Concern checklist and the Denmark checklist) were sourced from the Ministeriet for Grøn Trepart^24,26,48–52^. Finally, the European Union list of invasive alien species of Union Concern and its updates were also incorporated (Union Concern Checklist hereafter)^24,26,50–52^ was also considered.

All checklists were updated until December 2025.

All taxon names were standardised using the World Checklist of Vascular Plants (WCVP) using the Taxamatch algorithm, as implemented in the Taxonomic Name Resolution Service (TNRS) v 5.3.1^53–59^. This process yielded a preliminary checklist of invasive vascular plants for the Celtic Fringe (AISCelt checklist).

### Input data

Georeferenced records of vascular plants identified as invasive in the Celtic Fringe (i. e. belonging to the AISCelt checklist), including the Iberian Peninsula, France, the British Isles, and the Faroe Islands, were compiled from both national and international biodiversity databases. These included the Global Biodiversity Information Facility (GBIF) (https://www.gbif.org)^60–77^, iNaturalist filtering observations with research quality degree (https://www.inaturalist.org/)^78–130^, Tela-Botanica^131^, and the Inventaire National du Patrimoine Naturel (INPN)^36^. Occurrence data for Ireland were sourced from various datasets within the National Biodiversity Centre^132–138^. GBIF data were updated through April 2025.

Updates to these national checklists and data sources will be used for future updates of the dynamic version of this database.

### National AIS checklists and species status

Because the Celtic Fringe spans countries that also encompass other floristic regions, such as the Mediterranean Region, the preliminary AISCelt checklist was filtered to exclude species absent from the target subprovinces. After standardizing the occurrence data, we used occurrence records to confirm the presence of each taxon within the Celtic Fringe subprovinces.

Some species are native to one part of the Celtic Fringe but invasive in another. These were categorized as Native and Alien Invasive Species of the Celtic Fringe (NAIS). Identification of NAIS taxa relied on a combination of occurrence records, native range data from Plants of the World Online^139^, and regional floras and checklists: *Flora Iberica*^140^, the Atlas of the vascular flora of the Iberian Peninsula (AFLIBER)^22^, the Tracheophyta database of France métropolitaine^36,131^, the Botanical Society of Britain & Ireland and the BIFloraExplorer database^23,141^, Botany of the Faeröes^142–144^ and the latest revision of the Plant Communities of the Faroe Islands^145^. NAISCelt taxa were identified and validated using a combination of the occurrence data, native range data from POWO^139^, and regional floras and checklists^22,23,43,142,144–146^. During this validation process contradictory information between local checklists and POWO was resolved by favouring the local checklist sources of native status. When a taxon was absent in both the local checklists and POWO, it was considered as alien due to the absence of records. Finally, when a taxon was absent in both the local checklists and was labelled as “Introduced” in POWO, this was considered as a double confirmation of “Alien” status.

Each taxon occurrence was assigned a “native” status when it was within the species’ natural range, or “alien” when found outside its native range and/or listed as invasive in that country. Afterwards, we treated separately each country occurrences dataset to categorize the status of the NAIS taxa in each country. Thus, NAIS occurrences were treated as having “native” status when NAIS taxa were within their natural range and “alien” when the NAIS taxon was not native to the specific country and considered invasive in that country.

### Standardizing species occurrences

**Fig. 2:**
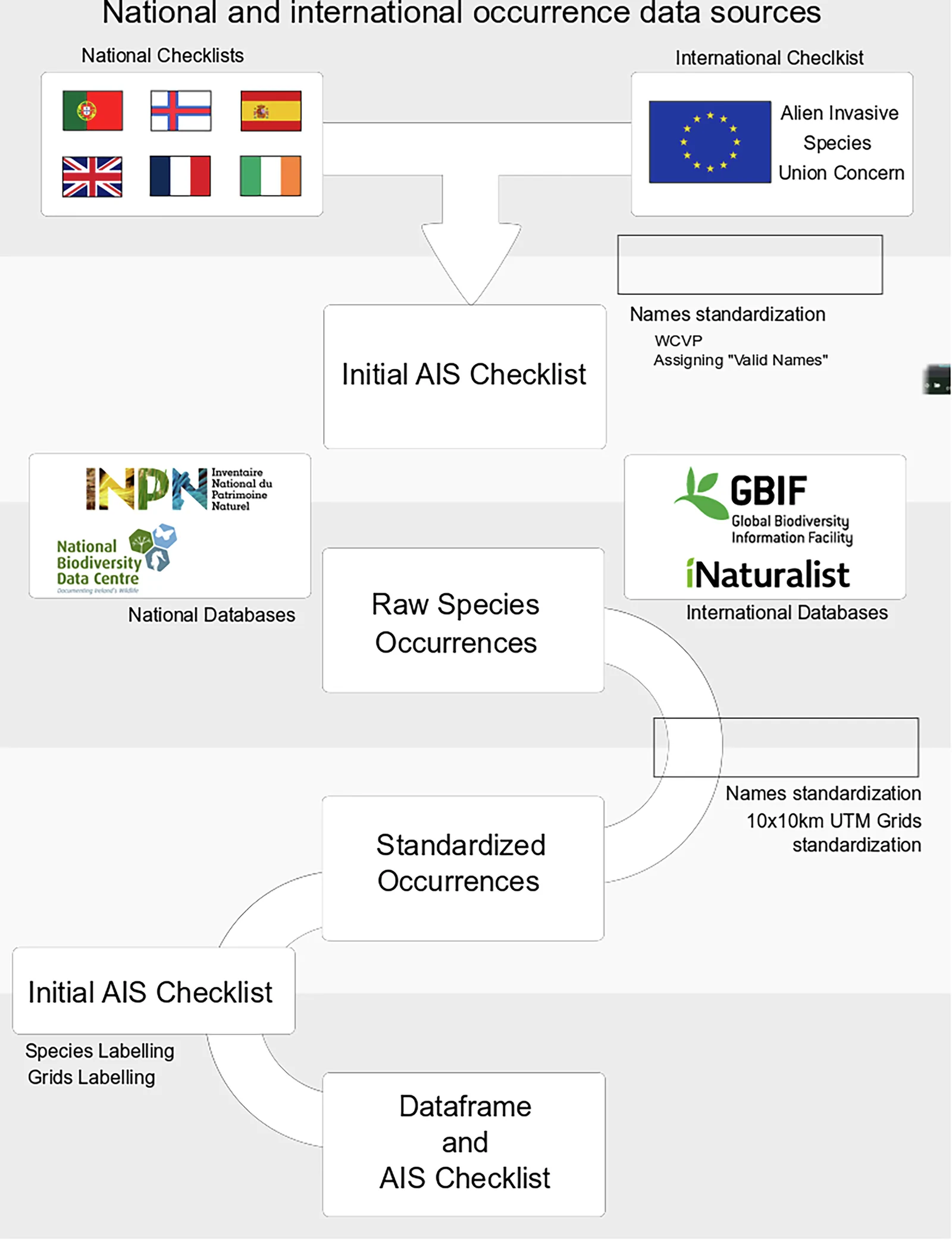
Scheme representing the process of building the Celtic Invasive Species database.

After assembling the preliminary AISCelt checklist, we generated a standardized georeferenced occurrence database at a 10 × 10 km UTM grid resolution (see Fig. 2). This 10 × 10 km resolution has proven effective in balancing the trade-off between geographic precision of occurrences and the loss of species records, and in mitigating the biases derived from the differing data mobilization policies^147,148^.

Both modern and historical herbarium records (i.e. some GBIF accessions) were considered as input data, discarding only records without associated coordinates. GBIF species records of cultivated individuals were also considered as input data, since the introduction of many plant AIS is closely related to ornamental or landscape management purposes^149^.

We took a conservative approach to species distribution griding accuracy by retaining records with coordinate uncertainty equal to or below 5,000 meters. Occurrence coordinates were assigned to the corresponding UTM 10 × 10 km grids using the mgrs Python package (version 1.4.3)^150^. These grids were filtered using the list of UTM 10 × 10 km grids contained within the target area, thus automatically removing records with erroneous coordinates (e.g. found in the ocean).

Taxon names were again standardised to WCVP-accepted names using the TNRS v 5.3.1 implementation of the Taxamatch algorithm^53–56,58,59,139^. Only occurrences matching the validated AISCelt checklist were retained, and duplicates were removed. This preliminary dataset was used to estimate species richness per grid and assess potential biases towards countries’ centroids or biodiversity institutions through a visual inspection. This process produced the final curated version of both the checklist and the occurrence dataset.

### Taxonomic, floristic and administrative grid data

Each taxon record includes its validated author name and genus (WCVP)^56,58,59^, family name (POWO backbone) and POWO taxon ID and URL^139^. Their superior taxonomic levels were obtained following the backbone of The Angiosperm Phylogeny Group (APG) IV^151^. Since our target taxonomic group is vascular plants (tracheophyta), we used this backbone to generate (1) a major phylum taxonomic category (‘Phylum’) which specifies whether the taxa belongs to Spermatophyta or Pteridophyta and (2) a major subphylum taxonomic category (‘Subphylum’) specifying if the taxa belongs to Angiosperms, Monilophyta or Gymnosperms^151^. Additionally, we used the APG IV backbone for taxa orders to label the POWO backbone valid family name of each taxa^139,151^.

Each taxon was annotated with its listed or non-listed status in both national invasive species checklists and the Union Concern list, and with its native or alien status within the Celtic Fringe. In the cases where national invasive species checklists listed genera instead of individual species, for example the Spanish checklist refers to “*Azolla* spp.”^32–35^ and the Portuguese to “*Acacia* spp.”^31^, all the species belonging to the genera were considered as listed in the national checklists. Taxon alien or native status within the Celtic Fringe and the individual countries was also annotated, and so was their AIS/NAIS denomination. Because the United Kingdom comprises areas in Great Britain and Ireland, the native/alien status of the NAIS occurring in Northern Ireland were treated separately following the same criteria as those of the Republic of Ireland.

The floristic subprovince classification was assigned according to the subprovince boundaries defined by Rivas-Martínez, Penas & Díaz^14^, Fernández Prieto *et al*.^16^ and Instituto Geográfico Nacional^30^. Each 10 × 10 km UTM grid was annotated accordingly.

Administrative data were extracted for each grid (except for the England smaller administrative units) using the osmdata R package version 0.2.5^152^, which retrieves information from OpenStreetMap data^153^. These data included the country (or the associated country of a self-governing territory), the official designation of Crown Dependencies or self-governing territory where applicable (United Kingdom and Denmark), the largest administrative units below the country level (Distrito of Portugal/Comunidad Autónoma/Régions/Provinces of Ireland/England Combined Authorities & England Combined County Authorities & England Councils & Scotland Council Areas & Wales Principal Areas & Northern Ireland Districts/Sýslas) and the second largest administrative unit beneath country or self-governing territory where applicable (Provincia/Département/County of Ireland/ County of England/Kommuna). The English first and second largest administrative units beneath country were obtained using the United Kingdom maps (level 4) provided by geodata R package version 0.6.6^154^ to generate the extanding Combined Authorities, Combined County Authorities and Councils and the sf R package version 1.0.20^155,156^ to locate the administrative units of the grids centroids.

Once obtained, the first and second largest administrative units beneath country were visually inspected against the 10 × 10 km UTM grid resolution to detect (1) the units that would be too small to be consistent with this resolution and (2) the grids which comprised a complete unit large enough to be consisting with the resolution, but that were labelled as another unit due to the location of the grid centroid. In the case of units too small, these were merged with one or more other units in the same geographic location and the label was changed to name combining all the names of the merged units linked by “&”. Thus, the smaller units are presented both in the occurrences and mpas as a merged unit. This affected mainly small England counties and Faroe Island Kommunas. This affected small England Boroughs. In the case of the Greater London boroughs, these were grouped using the London sub-regions established by the 2004 London Plan and its amendments^157–159^. When the units contained in a single grid that were not hit by the centroid of this, we corrected the labels by relocating the grid into this unit.

The presence of natural protected areas within each grid was also recorded. Centroid coordinates were cross-referenced with maps of nationally designated protected areas^160^, the Natura 2000 protected areas^161^ and the Pan-European network of protected sites (Emerald Networks)^162^. The sf R package v. 1.0–18^155,156^ was used to calculate the distance from each centroid to the nearest protected area. If the distance was 5,000 m or less, the presence of a protected area was recorded. Because this database focuses on vascular plants, polygons representing exclusively marine protected areas were discarded prior to distances estimation.

For each protected area, we recorded the name, national reserve type code, and, where available, additional details from the World Database of Protected Area (WDPA) (https://www.protectedplanet.net/en)^163–168^. These included the national and English designations of the area, associated WDPA identifiers (WDPA_ID), designation type, IUCN and United Nations Environment World Conservation Monitoring Centre (UNEP_WCMC) categories where applicable^169,170^, national protected-area type codes, management authority, government type, and ecosystem type.

Since different types of protected areas can overlap partially or entirely, we created three categories to describe protected-area presence within a grid: (1) presence of any protected area (national or Natura 2000), (2) presence of a Natura 2000 site, and (3) presence of a nationally designated protected area. Because partially overlapping Natura 2000 and nationally designated areas often have different names, we generated one column for nationally protected areas and another for Natura 2000 protected sites.

Finally, each taxon was annotated with its listed or non-listed status in both national invasive species checklists and the Union Concern list, as well as with its native or alien status within the Celtic Fringe.

## Data Record

All files forming the Celtic Invasive Plants database are available at EU Open Research Repository {Zenodo} (https://doi.org/10.5281/zenodo.18630660)^171^ as open access downloadable files. This database is structured into three folders: Database_and_Checklist, which includes the static versions of the database and checklist, Features and Maps.

The complete static version of the Celtic Invasive Plants database^171^ is provided in a downloadable tab separated CSV file (CIP_DB_APRIL_2025.csv). Each Celtic Invasive Plants database^171^ entry corresponds to a species occurrence and includes its 10 × 10 km UTM Grid name and centroid coordinates. The complete Celtic Invasive Plants checklist is provided as a separate file in which each entry corresponds with a different taxon (CIP_DB_APRIL_2025_CHECKLIST_&_VERIFICATION.csv).In both the database and checklist files, each species entry is associated to (1) its APG IV and WCVP-POWO valid taxonomic hierarchical groups (‘Phylum’, ‘Subphylum’, ‘Order’, ‘Family’, ‘Genus’, ‘Species with author’, ‘Taxa), (2) POWO taxon ID and direct URL of the taxon, (3) the Celtic Fringe origin status (‘Native’ vs ‘Alien’), (4) the Celtic Fringe Taxa Category (‘AIS’ vs ‘NAIS’), and (5) the listed status of the taxon in the Union List of Invasive Alien Species of Union Concern and in the local checklists. These two files treat the listed status in the local checklists in different manners: the Celtic Invasive Plants database^171^ stores this information in a single binary column (Officially_listed) which specifies if the taxa are listed in the area of the occurrence, while the complete Celtic Invasive Plants checklist treats each country official checklist(s) as individual categorical columns (Official_Status_{Country}) specifying the also the name under which the taxa have been listed and native or alien status if the taxon is classified as a NAIS. The Celtic Invasive Plants database^171^ file includes administrative and floristic information associated to each entry’s 10 × 10 km UTM Grid: (1) the country administrative levels of the grid (Country, Constituent Country or Crown Dependency, Administrative Unit level II and Administrative Unit Level III), (2) the presence and type of the protected areas and their associated metadata if these are present in the 10 × 10 km UTM Grid (e.g. Name of the protected area in English and in the native language, WDPA ID(s), type of majoritarian ecosystem…) and (3) floristic subprovince in which the centroid is located. The complete Celtic Invasive Plants checklist presents the “synonym” column detailing a list of synonyms of the valid WCVP valid taxa name found during the taxonomic standardization. The database and the complete checklist are accompanied by a comprehensive metadata file describing the information stored in each column, the type of variable and the different possible values (CIP_DB_APRIL_2025_DATA_DESCRIPTION.csv), which corresponds to Table 1, and its references (References_DATA_DESCRIPTION.txt).

**Table 1:** Description of the 76 different columns of the Celtic Invasive Plants database^171^ and the complete Celtic Invasive Plants checklist.

Column_name	Variable_Type	Description	Entry_treatment	Values	Dataset
Taxa	Categorical	Accepted Name of the Taxon according to the World Checklist of Vascular Plants (WCVP)^56,58,59^.	Several entry can have the same value.	Various (271x)	Celtic Invasive Plants database (CIP_DB_APRIL_2025.csv) & complete Celtic Invasive Plants checklist (CIP_DB_APRIL_2025_CHECKLIST_&_VERIFICATION.csv)
Phylum	Categorical	Major phylum of the Taxa according to the taxonomic backbone of the Angiosperm Phylogeny Group (APG) IV^151^	Same value for all entries with the same ‘Taxa’ value.	‘Spermatophyta’ OR ‘Pteridophyta’	Celtic Invasive Plants database (CIP_DB_APRIL_2025.csv) & complete Celtic Invasive Plants checklist (CIP_DB_APRIL_2025_CHECKLIST_&_VERIFICATION.csv)
Subphylum	Categorical	Subphylum of the Taxa according to the taxonomic backbone of the Angiosperm Phylogeny Group (APG) IV^151^ (Angiosperms/Monilophyta/Gymnosperms).	Same value for all entries with the same ‘Taxa’ value.	‘Angiosperms’ OR ‘Monilophyta’ OR ‘Gymnosperms’	Celtic Invasive Plants database (CIP_DB_APRIL_2025.csv) & complete Celtic Invasive Plants checklist (CIP_DB_APRIL_2025_CHECKLIST_&_VERIFICATION.csv)
Order	Categorical	Order of the Taxa according to the taxonomic backbone of the Angiosperm Phylogeny Group (APG) IV^151^	Same value for all entries with the same ‘Taxa’ value.	Various (39x)	Celtic Invasive Plants database (CIP_DB_APRIL_2025.csv) & complete Celtic Invasive Plants checklist (CIP_DB_APRIL_2025_CHECKLIST_&_VERIFICATION.csv)
Family	Categorical	Valid family according to the Plants of the World Online (POWO) backbone^139^	Same value for all entries with the same ‘Taxa’ value.	Various (79x)	Celtic Invasive Plants database (CIP_DB_APRIL_2025.csv) & complete Celtic Invasive Plants checklist (CIP_DB_APRIL_2025_CHECKLIST_&_VERIFICATION.csv)
Genus	Categorical	Valid genus according to the World Checklist of Vascular Plants (WCVP)^56,58,59^	Same value for all entries with the same ‘Taxa’ value.	Various (162x)	Celtic Invasive Plants database (CIP_DB_APRIL_2025.csv) & complete Celtic Invasive Plants checklist (CIP_DB_APRIL_2025_CHECKLIST_&_VERIFICATION.csv)
Species_with_Author	Categorical	Valid species name and author abbreviation according to the World Checklist of Vascular Plants (WCVP)^56,58,59^	Same value for all entries with the same ‘Taxa’ value.	Various (271x)	Celtic Invasive Plants database (CIP_DB_APRIL_2025.csv) & complete Celtic Invasive Plants checklist (CIP_DB_APRIL_2025_CHECKLIST_&_VERIFICATION.csv)
Taxa_ID	Categorical	Taxon the Plants of the World Online (POWO) ID^139^	Same value for all entries with the same ‘Taxa’ value.	Various (271x)	Celtic Invasive Plants database (CIP_DB_APRIL_2025.csv) & complete Celtic Invasive Plants checklist (CIP_DB_APRIL_2025_CHECKLIST_&_VERIFICATION.csv)
Taxa_URL	Categorical	Plants of the World Online (POWO)^139^ URL.	Same value for all entries with the same ‘Taxa’ value.	Various (271x)	Celtic Invasive Plants database (CIP_DB_APRIL_2025.csv) & complete Celtic Invasive Plants checklist (CIP_DB_APRIL_2025_CHECKLIST_&_VERIFICATION.csv)
Celtic_Fringe_Taxa_Category	Binary	Status of the specific taxon occurring in a certain UTM 10 × 10 Km grid. Taxa can be Native and Alien Invasive Species of the Celtic Fringe (NAIS), if they are native to certain subprovinces and countries of the Celtic Fringe of the European Atlantic Floristic Region and listed as AIS in other(s) or Alien Invasive Species of the Celtic Fringe (AIS), if they are listed as AIS in at list one of the countries (see CIP_DB_APRIL_2025_CHECKLIST_&_VERIFICATION.csv & CIP_DB_APRIL_2025_NAIS_VERIFICATION.csv).	Same value for all entries with the same ‘Taxa’ value.	‘AIS’ OR ‘NAIS’	Celtic Invasive Plants database (CIP_DB_APRIL_2025.csv) & complete Celtic Invasive Plants checklist (CIP_DB_APRIL_2025_CHECKLIST_&_VERIFICATION.csv)
Officially_listed	Binary	Whether the taxon is listed within Invasive Species Oficial Checklist of the country where the UTM 10 × 10 Km grid is located.	Same value for all entries with the same combination of ‘Taxa’ value and ‘Country’ value	‘YES’ OR ‘NO’	Celtic Invasive Plants database (CIP_DB_APRIL_2025.csv)
EU_Concern	Binary	Whether the taxa is listed within the EU Union Concern checklist^24,26,50–52^	Same value for all entries with the same ‘Taxa’ value.	‘YES’ OR ‘NO’	Celtic Invasive Plants database (CIP_DB_APRIL_2025.csv) & complete Celtic Invasive Plants checklist (CIP_DB_APRIL_2025_CHECKLIST_&_VERIFICATION.csv)
Celtic_Fringe_Origin	Binary	Whether the taxon is native of alien to the Celtic Fringe of the European Atlantic Floristic Region (see CIP_DB_APRIL_2025_CHECKLIST_&_VERIFICATION.csv & CIP_DB_APRIL_2025_NAIS_VERIFICATION.csv).	Same value for all entries with the same ‘Taxa’ value.	‘Alien’ OR ‘Native’	Celtic Invasive Plants database (CIP_DB_APRIL_2025.csv) & complete Celtic Invasive Plants checklist (CIP_DB_APRIL_2025_CHECKLIST_&_VERIFICATION.csv)
Local_Origin	Binary	Whether the taxon is native of alien to the country in which the occurrence is located (see CIP_DB_APRIL_2025_CHECKLIST_&_VERIFICATION.csv & CIP_DB_APRIL_2025_NAIS_VERIFICATION.csv) (Native/Alien).	Same value for all entries with the same combination of ‘Taxa’ value and ‘Country’ value	‘Alien’ OR ‘Native’ OR ‘Extinct’	Celtic Invasive Plants database (CIP_DB_APRIL_2025.csv)
Subprovince	Categorical	Subprovince of the Celtic Fringe of the European Atlantic Floristic Region acoording to the boundaries of Rivas-Martínez, Penas & Díaz^14^, Fernández Prieto *et al*.^16^ and Instituto Geográfico Nacional^30^ to which the centroid of UTM 10 × 10 Km grid belongs.	Same value for all entries with the same ‘UTM_grid’ value.	‘Cantabrian Atlantic’ OR ‘Orocantabrian’ OR ‘French Atlantic’ OR ‘Britannic’	Celtic Invasive Plants database (CIP_DB_APRIL_2025.csv)
UTM_grid	Categorical	UTM 10 × 10 Km grid name.	Several entry can have the same value.	Various (7843x)	Celtic Invasive Plants database (CIP_DB_APRIL_2025.csv)
Latitude	Numeric	Decimal coordinates of the centroid of the UTM 10 × 10 Km grid.	Same value for all entries with the same ‘UTM_grid’ value.	Various (3998x)	Celtic Invasive Plants database (CIP_DB_APRIL_2025.csv)
Longitude	Numeric	Decimal coordinates of the centroid of the UTM 10 × 10 Km grid.	Same value for all entries with the same ‘UTM_grid’ value.	Various (7638x)	Celtic Invasive Plants database (CIP_DB_APRIL_2025.csv)
Country	Categorical	Country to which the centroid decimal coordinates of the UTM 10 × 10 Km grid belongs.	Same value for all entries with the same ‘UTM_grid’ value.	‘France’ OR ‘Portugal’ OR ‘Spain/España’ OR ‘United Kingdom’ OR ‘Ireland/Éire’ OR ‘Denmark/Danmark/Danmørk’	Celtic Invasive Plants database (CIP_DB_APRIL_2025.csv)
Constituent_Country_OR_Crown_Dependency	Categorical	Only applicable to Denmark (Faroe Island) and the United Kingdom.	Same value for all entries with the same ‘UTM_grid’ value.	“England” OR ‘Wales/Cymru’ OR ‘Scotland/Alba’, Jersey/Jèrri’ OR ‘Bailiwick of Guernsey/Bailliage dé Guernési’ OR ‘Northern Ireland/Tuaisceart Éireann’ OR ‘Isle of Man/Ellan Vannin’ OR ‘Faroe Islands/Føroyar’	Celtic Invasive Plants database (CIP_DB_APRIL_2025.csv)
Admin_units_II	Categorical	This corresponds to the the largest administrative units beneath country or self-governing territory. Porugal: Distrito, Spain: Comunidad Autónoma, France: Régions, UK: England Combined Authorities & England Combined County Authorities & England Councils & Scotland Council Areas & Wales Principal Areas & Northern Ireland Districts, Ireland: Provinces of Ireland (Cúigí na hÉireann) and Faroe Islands: Sýsla.	Same value for all entries with the same ‘UTM_grid’ value.	Various (176x)	Celtic Invasive Plants database (CIP_DB_APRIL_2025.csv)
Admin_units_III	Categorical	Second largest administrative unit beneath country or self-governing territory where applicable Portugal: Districto, Spain: Provincia, France: Département, Ireland: County of Ireland, UK: Counties of England and Faroe Islands: Kommuna).	Same value for all entries with the same ‘UTM_grid’ value.	Various (235x)	Celtic Invasive Plants database (CIP_DB_APRIL_2025.csv)
Presence_Protected_Area	Binary	Presence of any nature protected area either nationally designated or belonging to the Natura 2000 network.	Same value for all entries with the same ‘UTM_grid’ value.	‘1’ (= present) OR ‘0’ (= not present)	Celtic Invasive Plants database (CIP_DB_APRIL_2025.csv)
Presence_Natura_2000	Binary	Presence of a protected area belonging to the Natura 2000 network.	Same value for all entries with the same ‘UTM_grid’ value. Only applicable to entry with a value of ‘1’ in the ‘Presence_Protected_Area’ column.	‘1’ (= present) OR ‘0’ (= not present)	Celtic Invasive Plants database (CIP_DB_APRIL_2025.csv)
Presence_National_Nature_Reserve	Binary	Presence of nationally designated protected area.	Same value for all entries with the same ‘UTM_grid’ value. Only applicable to entry with a value of ‘1’ in the ‘Presence_Protected_Area’ column.	‘1’ (= present) OR ‘0’ (= not present)	Celtic Invasive Plants database (CIP_DB_APRIL_2025.csv)
National_Nature_Reserve_Name	Categorical	Official name of a nationally designated protected area according to the World Database of Protected Area (WDPA) data of each country^163–168^	Same value for all entries with the same ‘UTM_grid’ value. Only applicable to entry with a value of ‘1’ in the ‘Presence_Protected_Area’ column.	Various (1704x)	Celtic Invasive Plants database (CIP_DB_APRIL_2025.csv)
Natura_2000_Name	Categorical	Official name of a Natura 2000 protected area according to the World Database of Protected Area (WDPA) data of each country^163–168^	Same value for all entries with the same ‘UTM_grid’ value. Only applicable to entry with a value of ‘1’ in the ‘Presence_Protected_Area’ column.	Various (1097x)	Celtic Invasive Plants database (CIP_DB_APRIL_2025.csv)
Special_Areas_of_Conservation_(Habitats_Directive)	Binary	Only applicable to protected areas belonging to the Natura 2000 Network. This columns specifies whether the protected area is classified as Special Areas of Conservation of the EU Habitats Directive^175^ according to the World Database of Protected Area (WDPA) (https://www.protectedplanet.net/en).	Same value for all entries with the same ‘Natura_2000_Name’ value. Only applicable to entry with a value of ‘1’ in the ‘Presence_Natura_2000 column.	‘YES’ OR ‘NO’	Celtic Invasive Plants database (CIP_DB_APRIL_2025.csv)
Special_Protection_Area_(Birds_Directive)	Binary	Only applicable to protected areas belonging to the Natura 2000 Network. This columns specifies whether the protected area is classified as Special Protection Area in the EU Birds Directive^176^ according to the World Database of Protected Area (WDPA) (https://www.protectedplanet.net/en)	Same value for all entries with the same ‘Natura_2000_Name’ value. Only applicable to entry with a value of ‘1’ in the ‘Presence_Natura_2000 column.	‘YES’ OR ‘NO’	Celtic Invasive Plants database (CIP_DB_APRIL_2025.csv)
Site_of_Community_Importance_(Habitats_Directive)	Binary	Only applicable to protected areas belonging to the Natura 2000 Network. This columns specifies whether the protected area is classified as Site of Community Importance of the EU Habitats Directive^175^ according to the World Database of Protected Area (WDPA) (https://www.protectedplanet.net/en)	Same value for all entries with the same ‘Natura_2000_Name’ value. Only applicable to entry with a value of ‘1’ in the ‘Presence_Natura_2000 column.	‘YES’ OR ‘NO’	Celtic Invasive Plants database (CIP_DB_APRIL_2025.csv)
National_Designation	Categorical	National category of the protected area in the native language of the country.	Same value for all entries with the same ‘Natura_2000_Name’ and/ or ‘National_Nature_Reserve_Name’value. Only applicable to entry with a value of ‘1’ in the ‘Presence_Protected_Area’ column.	Various & combinations (124x)	Celtic Invasive Plants database (CIP_DB_APRIL_2025.csv)
Designation_in_English	Categorical	National category of the protected area in English.	Same value for all entries with the same ‘Natura_2000_Name’ and/ or ‘National_Nature_Reserve_Name’value. Only applicable to entry with a value of ‘1’ in the ‘Presence_Protected_Area’ column.	Various & combinations (120x)	Celtic Invasive Plants database (CIP_DB_APRIL_2025.csv)
Designation_Type	Categorical	Type of management scope of the protected area according to the World Database of Protected Area (WDPA) (https://www.protectedplanet.net/en).	Same value for all entries with the same ‘Natura_2000_Name’ and/ or ‘National_Nature_Reserve_Name’value. Only applicable to entry with a value of ‘1’ in the ‘Presence_Protected_Area’ column.	‘Regional’ OR ‘National’ OR ‘International & combinations of these (7x)	Celtic Invasive Plants database (CIP_DB_APRIL_2025.csv)
IUCN_Cat	Numeric	International Union for Conservation of Nature (IUCN) protected area category^169,170^ according to the World Database of Protected Area (WDPA) (https://www.protectedplanet.net/en)	Same value for all entries with the same ‘Natura_2000_Name’ and/ or ‘National_Nature_Reserve_Name’value. Only appliable to entry with a value of ‘1’ in the ‘Presence_Protected_Area’ column.	‘Ia’ OR ‘II’ OR ‘III’ OR ‘IV’ OR ‘V’, ‘VI’ OR ‘Not Reported’ OR ‘Not Assigned’ OR ‘Not Applicable’& combinations of these (14x)	Celtic Invasive Plants database (CIP_DB_APRIL_2025.csv)
UNEP_WCMC_Cat	Numeric	United Nations Environnment World Conservation Monitoring Centre (UNEP_WCMC) category. This is only applied to Ramsar and World Heritage sites.	Same value for all entries with the same ‘Natura_2000_Name’ and/ or ‘National_Nature_Reserve_Name’value. Only applicable to entry with a value of ‘1’ in the ‘Presence_Protected_Area’ column.	Various & combinations (29x)	Celtic Invasive Plants database (CIP_DB_APRIL_2025.csv)
Reserve_Ecosystem_type	Categorical	General type(s) of ecosystms forming the protecte area according to the World Database of Protected Area (WDPA) (https://www.protectedplanet.net/en).	Same value for all entries with the same ‘Natura_2000_Name’ and/ or ‘National_Nature_Reserve_Name’value. Only applicable to entry with a value of ‘1’ in the ‘Presence_Protected_Area’ column.	‘Terrestrial’ OR ‘Coastal’ OR ‘Marine’ OR Combinations of these (8x)	Celtic Invasive Plants database (CIP_DB_APRIL_2025.csv)
Governance_Type	Categorical	Type of governance(s) according to the World Database of Protected Area (WDPA) (https://www.protectedplanet.net/en).	Same value for all entries with the same ‘Natura_2000_Name’ and/ or ‘National_Nature_Reserve_Name’value. Only applicable to entry with a value of ‘1’ in the ‘Presence_Protected_Area’ column.	Various & combinations (15x)	Celtic Invasive Plants database (CIP_DB_APRIL_2025.csv)
Management_Authority	Categorical	Management Authority according to the World Database of Protected Area (WDPA) (https://www.protectedplanet.net/en).	Same value for all entries with the same ‘Natura_2000_Name’ and/ or ‘National_Nature_Reserve_Name’value. Only applicable to entry with a value of ‘1’ in the ‘Presence_Protected_Area’ column.	Various & combinations (93x)	Celtic Invasive Plants database (CIP_DB_APRIL_2025.csv)
_WDPA_ID columns (38x)	Numeric	Several different columns depending on the designation categories of each country. Typically named as [Designation_English]_WDPA_ID. These columns present the World Database of Protected Area (WDPA) (https://www.protectedplanet.net/en) identification numbers of the different designations of a protected area. A protected area can present various different designations and WDPA_IDs.	Same values for all entries with the same ‘Natura_2000_Name’ and/ or ‘National_Nature_Reserve_Name’value. Only applicable to entry with a value of ‘1’ in the ‘Presence_Protected_Area’ column.	Various & combinations	Celtic Invasive Plants database (CIP_DB_APRIL_2025.csv)
Official_Status_Portugal	Categorical	Listed status of the taxon within the AIS checklist provided by the Ministry Environment and Energetic Transition of the Portuguese Government^31^. If the taxon entry has a value of NAIS in the Celtic_Fringe_Taxa_Category column, the Native/Alien status is also specified. The name under which the taxon was listed is also provided.	A value per taxon entry.	Combination of values: ‘Listed’ (= the taxon is listed), ‘Native’ (= the NAIS is native to Portugal), ‘Alien’ (= the NAIS is alien to Portugal). [NAME]: specifies the name under which the taxon was listed	Complete Celtic Invasive Plants checklist (CIP_DB_APRIL_2025_CHECKLIST_&_VERIFICATION.csv)
Official_Status_Spain	Categorical	Listed status of the taxon within the AIS checklist provided by the Spanish Ministry for the Ecological Transition^32–35^. If the taxon entry has a value of NAIS in the Celtic_Fringe_Taxa_Category column, the Native/Alien status is also specified. The name under which the taxon was listed is also provided.	A value per taxon entry.	Combination of values: ‘Listed’ (= the taxon is listed), ‘Native’ (= The NAIS is native to Spain), ‘Alien’ (= The NAIS is alien to Spain). [NAME]: specifies the name under which the taxon was listed	Complete Celtic Invasive Plants checklist (CIP_DB_APRIL_2025_CHECKLIST_&_VERIFICATION.csv)
Official_Status_France	Categorical	Listed status of the taxon within the AIS checklist provided by the Inventaire National du Patrimoine Naturel (INPN) and the French Ministry of Ecological Transition^36–38^.If the taxon entry has a value of NAIS in the Celtic_Fringe_Taxa_Category column, the Native/Alien status is also specified. The name under which the taxon was listed is also provided.	A value per taxon entry.	Combination of values: ‘Listed’ (= the taxon is listed), ‘Native’ (= The NAIS is native to France), ‘Alien’ (= The NAIS is alien to France). [NAME]: specifies the name under which the taxon was listed	Complete Celtic Invasive Plants checklist (CIP_DB_APRIL_2025_CHECKLIST_&_VERIFICATION.csv)
Official_Status_Ireland	Categorical	Listed status of the taxon within the AIS checklist provided by the National Biodiversity Data Centre of Ireland^44,45^ and the Government official list and updates^46,47^. If the taxon entry has a value of NAIS in the Celtic_Fringe_Taxa_Category column, the Native/Alien status is also specified The name under which the taxon was listed is also provided.	A value per taxon entry.	Combination of values: ‘Listed’ (= the taxon is listed), ‘Native’ (= The NAIS is native to Ireland), ‘Alien’ (= The NAIS is alien to Ireland). [NAME]: specifies the name under which the taxon was listed	Complete Celtic Invasive Plants checklist (CIP_DB_APRIL_2025_CHECKLIST_&_VERIFICATION.csv)
Official_Status_UK	Categorical	Listed status of the taxon within the AIS checklist provided by the Natural England report^40^, the Great Britain Non-Native Species Secretariat (NNSS)^41^ and the comprehensive summary of all UK local list provided by the Royal Horticultural Society (RHS)^42,43^. If the taxon entry has a value of NAIS in the Celtic_Fringe_Taxa_Category column, the Native/Alien status is also specified. The name under which the taxon was listed is also provided.	A value per taxon entry.	Combination of values: ‘Listed’ (= the taxon is listed), ‘Native’ (= The NAIS is native to the United Kingdom (UK)), ‘Alien’ (= The NAIS is alien to United Kingdom (UK)). [NAME]: specifies the name under which the taxon was listed.	Complete Celtic Invasive Plants checklist (CIP_DB_APRIL_2025_CHECKLIST_&_VERIFICATION.csv)
Official_Status_Denmark	Categorical	Listed status of the taxon within the the two legally binding AIS checklist (the Union Concern checklist and the Denmark checklist)provided by the Ministeriet for Grøn Trepart^24,26,48–52^. If the taxon entry has a value of NAIS in the Celtic_Fringe_Taxa_Category column, the Native/Alien status is also specified. The name under which the taxon was listed is also provided.	A value per taxon entry.	Combination of values: ‘Listed’ (= the taxon is listed), ‘Native’ (= The NAIS is native to the Faroe Islands (Denmark)), ‘Alien’ (= The NAIS is alien to the Faroe Islands (Denmark)). [NAME]: specifies the name under which the taxon was listed	Complete Celtic Invasive Plants checklist (CIP_DB_APRIL_2025_CHECKLIST_&_VERIFICATION.csv)
Synonyms	List of values	List of queries uploaded to Taxonomic Name Resolution Service (TNRS) v 5.3.1 that retrieved the same valid Species Name according to the World Checklist of Vascular Plants (WCVP)^53–56,59,139^	A list per taxon entry.	Various (271x)	Complete Celtic Invasive Plants checklist (CIP_DB_APRIL_2025_CHECKLIST_&_VERIFICATION.csv)

In addition, the results and the references used during the NAIS validation process are summarized in two files (CIP_DB_APRIL_2025_NAIS_VERIFICATION.csv and Reference_NAIS_TAXA_VALIDATION.txt). In this file each NAIS taxa represents a single entry and their native or alien status reported by each country column (Status_{country}). The information obtained from each country reference is displayed specifying the category or categories used by these sources.

These basic files of the database are complemented by a series of feature files specifying (1) the unique values per column of the whole database (CIP_DB_APRIL_2025_UNIQUE_VALUES.csv), (2) a csv file of the unique values per country (6x) (unique_values{Country}.csv) and (3) a file detailing the all the included features of each Protected Area included in the target area (CIP_DB_APRIL_2025_PROTECTED_AREAS.csv). The modifications generated by the process of relocation or merging of the Admin units II and/or Admin units III information can be verified, as we included a file detailing the initial values for 10 × 10 km UTM grids, the type of modification and the resulting value (CIP_DB_APRIL_2025_Grids_merges_ and_relocations.csv).

A complete map containing the 10 × 10 km UTM grids polygons and the polygons used to label the occurrence’s floristic areas, political administrative units and protected areas has been generated (ALL_MAPS_CIP_2025.gpkg). These files contain 10 layers: Celtic_Fringe, Subprovinces, Countries, UK_Crown_dependencies, Admin_Units_II, Admin_Units_III, Parks_and_Reserves, Grids_Zone_29, Grids_Zone_30 and Grids_Zone_31. The Celtic_Fringe and Subprovinces layers were built by modifying the EEA map of the Biogeograhical regions in Europe^13^ using our target area outer and inner boundaries. The Countries, UK_Crown_dependencies, Admin_Units_II, Admin_Units_III layers were obtained by merging different polygons of the maps provided by geodata R package version 0.6.6^154^. The Parks_and_Reserves layer was generated by selecting the nationally designed protected areas^160^, the Natura 2000 protected areas^161^ and the Pan-European network of protected sites (Emerald Networks)^162^ polygons of the protected areas present in the database target area and joining them in a unique layer. We labelled the polygons by adding the binary columns Natura_2000 and National_Nature_Reserve. The Grids_zone_{UTM zone} layers were built by projecting the 10 × 10 km UTM grids using the coordinates of the grids centroids and the utm (version 0.8.1) and geopandas (version 0.8.1) Python packages^172,173^. We associated the UTM grid name, Celtic Fringe subprovince the political administrative units and protected areas information to each polygon of these layers.

## Technical Validation

The quality and reliability of the Celtic Invasive Plants database^171^ are supported by three core components: (1) the accuracy of taxonomic standardization, (2) the consistency of species distribution data, and (3) the verification of species’ invasive status across different national and international checklists.

We associated the listed status and names of each species across all AIS Checklists used to build the database, allowing alien species not included in any checklist to be readily identified and removed. Valid names were manually reviewed, and the POWO database was consulted to determine the native/alien status of each taxon within the Celtic Fringe and its constituent countries. Where POWO data were unclear, local floras and checklists were consulted. Together, these steps ensured that the three principal aspects affecting database quality were thoroughly cross-checked and validated throughout the process.

## Usage Notes

The Celtic Invasive Plants database^171^ was developed to support a wide range of applications in invasion ecology, conservation planning, and biodiversity policy. The dataset enables the rapid extraction of invasive species lists for specific regions within the Celtic Fringe, whether defined by floristic subprovince, national or subnational administrative units, or designated natural reserves. Such filtered inventories can inform the design of management strategies, optimize early detection and rapid response systems, and prioritize areas for monitoring and control.

The dataset also allows researchers and policymakers to compare the listed status of taxa across national checklists and the EU Union Concern list. This comparison facilitates the identification of invasive species that, although not officially recognized in some countries, may represent significant threats based on their confirmed presence and invasive behavior in neighboring regions with similar ecological conditions.

Beyond regulatory applications, the database supports broader ecological and biogeographic analyses. The inclusion of floristic subprovince classification enables the exploration of spatial variation in invasion patterns across distinct ecological zones. Meanwhile, the harmonized taxonomic backbone ensures compatibility with other regional or global biodiversity datasets, supporting integration into meta-analyses, modelling efforts, and conservation assessments.

To accommodate future research needs, the authors plan to maintain and periodically update the dataset. Users are encouraged to report any errors, new data, or proposed corrections to the corresponding author. Updates will incorporate new occurrence records, taxonomic changes, and revised species statuses as national or international lists are amended.

The grid and taxa data also allow users to generate full AIS inventories focusing on different administrative divisions. For example, the database can be used to compile an inventory of EU Concern taxa (EU_Concern = “YES”) occurring in a particular country, lower administrative units or protected area. Similarly, the database can help identify unlisted species that may pose a local threat (Officially_listed = ‘NO’) or NAISCelt taxa (Celtic_Fringe_Taxa_Category = ‘NAIS’).

Overall, this database provides a foundational tool for tracking, understanding, and mitigating the impacts of alien invasive vascular plants in one of Europe’s most ecologically distinctive regions^171^.

## Data Availability

The static and peer-viewed version of this database (Version v4, Published February 13, 2026) deposited at Zenodo will remain fixed and publicly available at https://doi.org/10.5281/zenodo.18630660^171^, reflecting the dataset as it existed at the time of publication of this data descriptor. This database is shared under the Creative Commons Attribution 4.0 International (CC BY 4.0) and the Creative Commons licence. Images of the target area and the database building process are also available at this website.

## Code availability

We have developed an R package based on the database, available on GitHub (https://github.com/Cgt93/CelticInvasivePlantsdb)^174^. This package is meant to serve as a dynamic and evolving representation of the data linked to the latest updates and revisions. Future releases of the package will also include biogeographic data relevant to AIS and their management.

The static version of the database deposited at Zenodo will remain fixed, reflecting the dataset as it existed at the time of publication of this data descriptor.
